# Analysis of the Correlation/Agreement of Maternal–fetal Doppler Parameters in Normal and Growth-Restricted Fetuses

**DOI:** 10.1055/s-0041-1741453

**Published:** 2022-02-25

**Authors:** Ederlei Munhoz Pinsuti, Rafael Frederico Bruns, Jaime Kulak Júnior, Newton Sérgio Carvalho, Dênis José Nascimento, Ana Cristina Perez Zamarian, Edward Araújo Júnior

**Affiliations:** 1 Department of Gynecology and Obstetrics, Universidade Federal do Paraná, Curitiba, PR, Brazil; 2 Department of Obstetrics, Escola Paulista de Medicina, Universidade Federal de São Paulo, São Paulo, SP, Brazil

**Keywords:** fetal growth restriction, doppler, cerebroplacental ratio, agreement, correlation, restrição do crescimento fetal, doppler, relação cérebro-placentária, concordância, correlação

## Abstract

**Objective** To assess the degree of correlation/agreement of maternal–fetal Doppler parameters between normal and growth-restricted fetuses (fetal growth restriction [FGR]).

**Methods** The present observational and retrospective study included 274 singleton pregnancies. The following maternal–fetal Doppler parameters were assessed: uterine artery (UAt), umbilical artery (UA), middle cerebral artery (MCA), cerebroplacental ratio (CPR), and umbilical–cerebral ratio (U/C). The assessment of FGR was based on the Figueiras and Gratacós 9 criteria. Spearman correlation coefficients were estimated to assess the correlation between resistance (RI) and pulsatility (PI) indices of Doppler parameters. The agreement between two Doppler parameters was assessed by the Kappa coefficient.

**Results** In total, 502 Doppler examinations were included, and FGR was observed in 19 out of 274 fetuses. A strong correlation was observed between RI and PI of UAt, UA, and MCA in all of the samples ( *p* < 0.001). Of the 502 Doppler examinations, there was agreement between U/C and CPR percentiles for 480 (95.6%) and disagreement for 22 (4.4%), with Kappa coefficient of 0.26, thereby corresponding to weak agreement. Of the 68 cases with estimated fetal weight ≤ 9 ^th^ percentile (small for gestational age [SGA]), there was agreement between U/C > 1.0 and CPR < 5 ^th^ percentile in 61 (88.4%) and disagreement in 7 (5.8%) with Kappa coefficient of 0.49, thereby corresponding to moderate agreement.

**Conclusion** Strong correlation was observed among RI and PI UAt, UA, and MCA Doppler examinations in the present study; however, weak agreement was observed between U/C and CPR in the normal and FGR fetuses. In SGA, U/C and CPR demonstrated moderate agreement.

## Resumo

**Objetivo** Avaliar o grau de correlação/concordância dos parâmetros Doppler materno-fetal entre fetos normais e com restrição do crescimento (restrição de crescimento fetal [RCF]).

**Métodos** O presente estudo observacional e retrospectivo incluiu 274 gestações únicas. Os seguintes parâmetros Doppler materno-fetal foram avaliados: artéria uterina (AUt), artéria umbilical (AU), artéria cerebral média (ACM), razão cérebro-placentária (RCP) e razão umbilical-cerebral (U/C). A avaliação da RCF baseou-se nos critérios de Figueiras e Gratacós. 9 Os coeficientes de correlação de Spearman foram estimados para avaliar a correlação entre os índices de resistência (IR) e pulsatilidade (IP) dos parâmetros Doppler. A concordância entre dois parâmetros do Doppler foi avaliada pelo coeficiente Kappa.

**Resultados** No total, 502 exames Doppler foram incluídos e RCF foi observado em 19 de 274 fetos. Observou-se forte correlação entre IR e IP da AUt, AU e ACM em todas as amostras ( *p* < 0,001). Dos 502 exames Doppler, houve concordância entre os percentis U/C e RCP para 480 (95,6%) e discordância para 22 (4,4%), com coeficiente Kappa de 0,26, correspondendo a concordância fraca. Dos 68 casos com peso fetal estimado ≤ 9° (pequeno para a idade gestacional [PIG]), houve concordância entre U/C > 1,0 e RC *p* < 5 ^o^ percentil em 61 (88,4%) e discordância em 7 (5,8%) com coeficiente Kappa de 0,49, correspondendo a concordância moderada.

**Conclusão** Forte correlação foi observada entre o IR e IP dos exames Doppler AUt, AU e ACM no presente estudo; entretanto, fraca concordância foi observada entre U/C e RCP em fetos normais e com RCF. Nos PIG, U/C e RCP demonstraram concordância moderada.

## Introduction

Fetal growth restriction (FGR) occurs when the conceptual product does not reach its intrauterine growth and development potential. However, in the clinical practice, FGR is difficult to define, and, to date, there is no gold standard method for its diagnosis. 1 Fetal growth restriction can be secondary to numerous conditions that include congenital malformations, chromosomal disorders, and intrauterine infections; however, most cases of FGR occur as a consequence of placental insufficiency that can lead to fetal hypoxia. 2

One of the first investigations regarding the clinical importance of FGR was performed by Lubchenco et al., 3 who determined the fetal weight in relation to gestational age at the time of delivery. This analysis resulted in an increase in perinatal morbidity and mortality in newborns weighing < 10 ^th^ percentile for gestational age. However, the definition of FGR using only the estimated fetal weight (EFW) < 10 ^th^ percentile would also encompass fetuses small for gestational age (SGA), and a distinction between restricted (those at high risk of perinatal complications) and SGA fetuses is necessary. 4

The society of Maternal-Fetal Medicine states that the prenatal detection of FGR can improve the perinatal outcome through appropriate fetal monitoring and optimization of the time of delivery. 5 Doppler has become an essential tool in the diagnosis and management of FGR. Some Doppler indices can be used to evaluate the waveform of both maternal and fetal vessels: resistance index (RI), represented by the systole-diastole/integral mean velocity of the spectral area, and the pulsatility index (PI), represented by the systole-diastole/systole. When RI is used, the Doppler waveform is represented only on a scale of 0 to 1 and has been reported to have a linear relationship with gestational age. In comparison, it is believed that the use of the PI allows continuous waveform analysis over a more extensive range of waveform patterns in addition to having a quadratic relationship with gestational age. 6

The Doppler assessment in FGR is based on the assessment of fetal well-being by examining the compensatory signs triggered by hypoxemia in the fetal circulation. 7 In the PORTO study, regardless of EFW or abdominal circumference (AC), the strongest and most significant association with adverse perinatal outcomes in the low-risk population was found when umbilical artery (UA) Doppler was altered. The authors suggest that EFW < 3 ^rd^ percentile or the combination of EFW < 10 ^th^ percentile with abnormal UA Doppler represent an increased risk of any adverse perinatal outcome or admission to the neonatal intensive care unit (ICU) compared with EFW or AC < 10 ^th^ percentile but with normal UA Doppler. 8

In the last decade, other factors that could help differentiate between SGA and FGR have been investigated; when altered, these parameters were associated with adverse perinatal outcomes. Estimated fetal weight or AC < 3 ^rd^ percentile, uterine artery (UtA) Doppler > 95 ^th^ percentile, middle cerebral artery (MCA) Doppler < 5 ^th^ percentile, and alteration in cerebroplacental ratio (CPR) were associated with adverse perinatal outcomes in low-weight fetuses. In the current FGR concept, UA Doppler should no longer be used as a single standard to determine diagnosis and prognosis. According to the criteria of Figueiras and Gratacós 9 FGR can be defined through the EFW < 3 ^rd^ percentile or the EFW between the 3 ^rd^ and 10 ^th^ percentile associated with altered maternal-fetal Doppler parameters.

Currently, the FGR is divided into early- and late-onset, with a cutoff of 32 weeks of pregnancy. However, this division does not represent only the gestational age at the diagnosis, but two entities with different natural histories and distinct biochemical, histological, and clinical characteristics. 7

In this context, we decided to investigate the behavior of maternal-fetal Doppler parameters and the CRP in fetuses with FGR and to evaluate the degree of agreement/correlation of maternal-fetal Doppler parameters between normal and FGR fetuses.

## Methods

The present observational and retrospective study was conducted in a private clinic of fetal medicine and at the Department of Gynecology and Obstetrics of the Universidade Federal do Paraná (UFPR, in the Portuguese acronym), Curitiba, state of Paraná, Brazil, between July 2017 and May 2019. The present study was approved by the Ethics Committee of UFPR, and consent form was not necessary as it was a retrospective study.

In total, 502 obstetrical ultrasound examinations with maternal-fetal Doppler parameters of 274 pregnant women were analyzed in the present study. The criteria of Figueiras and Gratacós were considered to evaluate the occurrence of FGR. 9 The sample inclusion criteria were singleton pregnancies from 24 weeks, considered to be at low risk or with FGR. The gestational age was determined by the last menstrual period and confirmed by ultrasound examination performed until 13 + 6 weeks of gestation using the crown-rump length.

The ultrasound examinations were performed by 10 doctors specialized in fetal medicine, who used the Voluson 730 PRO (General Electric Healthcare Zipf, Austria) and Accuvix V10 (Samsung-Medison, Seoul, South Korea) apparatus.

Data were collected regarding the gestational age at ultrasound examination, EFW with its percentile in the respective ultrasound examination, fetal abnormalities observed on the obstetric ultrasound examinations, and findings from the Doppler examination. Calculations were made to assess the respective percentiles of the RI and PI observed in the UtA, UA, MCA, and CPR with the Fetal ID calculator, v.2017 found on the Fetal Medicine Web site of Barcelona ( https://medicinafetalbarcelona.org/calc ). The CPR was obtained by dividing the PI of the MCA by the PI of the UA. Additionally, the umbilical-cerebral ratio (U/C) was obtained by dividing the RI of the UA by the RI of the MCA.

To assess the occurrence of FGR, the following criteria, according to Figueiras and Gratacós, 9 were considered: EFW < 3 ^rd^ or between the 3 ^rd^ and 9 ^th^ percentiles according to the table by Hadlock et al. 10 Additionally, the criteria also included at least 1 of the following conditions: UAt Doppler > 95 ^th^ , UA Doppler > 95 ^th^ , MCA Doppler < 5 ^th^ , and CPR < 5 ^th^ percentile. The U/C was considered altered if it was > 1.0. 11

The sample size was calculated to estimate the percentage of FGR. Considering an estimate of 6.8% for this percentage (Figueras et al.), 12 a sample of 271 fetuses would be sufficient to estimate this parameter with a margin of error of 3% and 95% confidence interval (CI).

The results of quantitative variables were described by means, standard deviations (SDs), medians, and ranges. Categorical variables were described by frequencies and percentages. A 95% CI was presented for determining the percentage of FGR. Spearman correlation coefficients were estimated to assess the correlation between PI and RI of maternal-fetal Doppler parameters. The agreement between two Doppler parameters was assessed by estimating the Kappa coefficient. The values of *p* < 0.05 indicated statistical significance. The data were analyzed using IBM SPSS Statistics for Windows, version 20.0 (IBM Corp., Armonk, NY, USA).

## Results

The present analysis was based on the data from 274 pregnant women who underwent Doppler examinations between 1 and 7 times from 24 weeks of gestation; moreover, 136 (49.6%) pregnant women underwent Doppler examination only 1 time. In total, 502 Doppler examinations were included in the study, and FGR was observed in 19 of 274 fetuses, with 4 early-onset (< 32 weeks) and 15 late-onset cases of FGR (≥ 32 weeks). Hence, it was estimated that the percentage of FGR was equal to 6.9% with a 95%CI (3.9–9.9%). Of the 274 pregnant women, 11 (4%) had any EFW < 3 ^rd^ percentile, 28 (10.2%) had any EFW assessment between the 3 ^rd^ and 9 ^th^ percentiles, and 235 (85.8%) had EFW ≥ 10 ^th^ percentile. Table 1 shows the descriptive analysis of all the maternal-fetal Doppler parameters.

**Table 1:** Descriptive analysis of maternal–fetal Doppler parameters

Doppler parameter	GA (weeks)	*n*	Mean	Median	Minimum	Maximum	Standard deviation
RI Right UAt	≤ 28	51	0.51	0.49	0.22	1.71	0.20
	28.1–32	112	0.49	0.48	0.23	0.85	0.10
	32.1–36	178	0.47	0.46	0.27	0.81	0.10
	> 36	161	0.45	0.44	0.08	0.77	0.10
PI Right UAt	≤ 28	51	0.77	0.73	0.28	2.17	0.30
	28.1–32	112	0.77	0.71	0.27	2.37	0.31
	32.1–36	178	0.71	0.67	0.34	1.71	0.24
	> 36	161	0.67	0.63	0.32	1.77	0.20
RI Left UAt	≤ 28	51	0.52	0.52	0.35	0.89	0.10
	28.1–32	112	0.49	0.48	0.30	0.74	0.09
	32.1–36	178	0.47	0.47	0.17	0.76	0.09
	> 36	161	0,45	0.44	0.24	0.78	0.09
PI Left UAt	≤ 28	51	0.80	0.77	0.45	1.65	0.22
	28.1–32	112	0.75	0.70	0.36	1.79	0.23
	32.1–36	178	0.71	0.68	0.24	1.90	0.23
	> 36	161	0.67	0.61	0.30	1.70	0.21
PI UA	≤ 28	51	1.14	1.14	0.57	1.89	0.20
	28.1–32	112	1.01	1.02	0.61	1.67	0.19
	32.1–36	178	0.90	0.89	0.55	1.48	0.17
	> 36	161	0.85	0.84	0.45	1.22	0.15
Percentile UA	≤ 28	51	45.69	46.00	3.00	99.00	20.41
	28.1–32	112	46.16	46.00	5.00	98.00	20.34
	32.1–36	178	43.92	41.00	8.00	96.00	20.06
	> 36	161	41.82	40.00	6.00	84.00	17.85
RI UA	≤28	51	0.70	0.70	0.45	0.88	0.07
	28.1–32	112	0.64	0.65	0.45	0.83	0.07
	32.1–36	178	0.59	0.60	0.43	0.79	0.07
	> 36	161	0.57	0.57	0.38	0.73	0.06
PI MCA	≤28	51	2.21	2.22	1.43	3.09	0.45
	28.1–32	112	2.17	2.13	0.86	3.65	0.51
	32.1–36	178	1.93	1.88	0.73	4.30	0.47
	>36	161	1,60	1.51	0.84	2.60	0.33
RI MCA	≤ 28	51	0.89	0.88	0.74	1.00	0.08
	28.1–32	112	0.88	0.87	0.57	1.06	0.08
	32.1–36	178	0.83	0.83	0.62	1.04	0.07
	> 36	161	0.77	0.76	0.55	1.00	0.07
CPR	≤ 28	51	1.98	1.93	1.21	3.45	0.50
	28.1–32	112	2.20	2.15	0.51	4.15	0.60
	32.1–36	178	2.20	2.16	0.69	3.98	0.58
	> 36	161	1.94	1.87	1.15	5.04	0.56
Percentile CPR	≤28	51	50.37	56.00	4.00	99.00	30.98
	28.1–32	112	53.04	51.50	1.00	99.00	31.08
	32.1–36	178	52.86	53.00	1.00	99.00	30.04
	> 36	161	43.07	38.00	3.00	99.00	30.07
U/C	≤ 28	51	0.79	0.80	0.58	0.99	0.10
	28.1–32	112	0.74	0.73	0.45	1.46	0.11
	32.1–36	178	0.72	0.71	0.51	1.08	0.10
	> 36	161	0.74	0.75	0.46	0.95	0.10
Percentile MCA	≤ 28	51	59.47	67.00	4.00	99.00	31.72
	28.1–32	112	56.79	60.00	1.00	99.00	32.66
	32.1–36	178	52.30	53.50	1.00	99.00	30.11
	> 36	161	48.09	46.00	1.00	99.00	29.16

Abbreviation: CPR, cerebroplacental ratio; GA, gestational age; MCA, middle cerebral artery; PI, pulsatility index; RI, resistance index; U/C, umbilical–cerebral ratio; UA, umbilical artery; UAt, uterine artery.

**Fig. 1:**
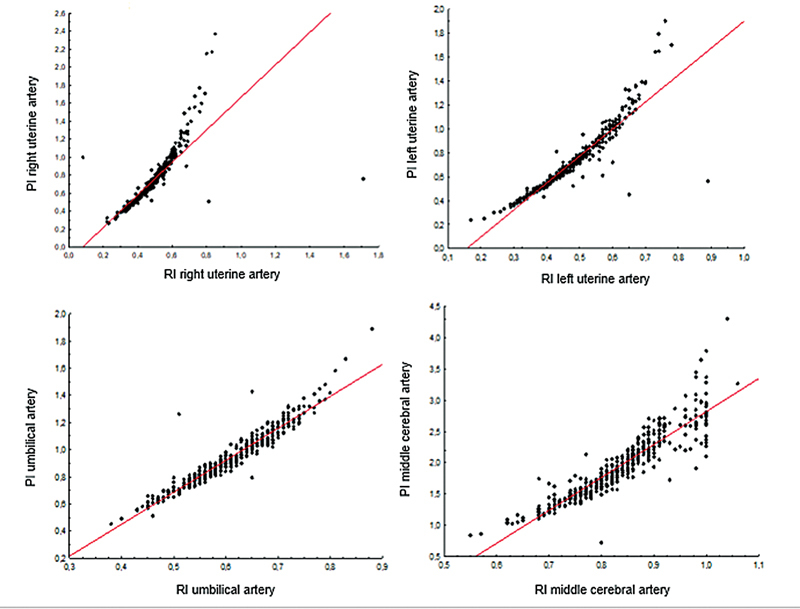
Correlation between resistance (RI) and pulsatility (PI) indices of maternal–fetal Doppler parameters.

Table 2 shows the correlation between RI and PI of UtA, UA, and MCA of all Doppler examinations. According to the Spearman correlation coefficient, there was an expressive and significant correlation between the Doppler parameters ( *p* < 0.001) ( Fig. 1 ).

**Table 2:** Correlation between maternal–fetal Doppler parameters

Variables	*n*	Spearman's correlation coefficient	*p-value*
RI Right UAt versus PI Right UAt	502	0.97	< 0.001
RI Left UAt versus PI Left UAt	502	0.97	< 0.001
RI UA versus PI UA	502	0.97	< 0.001
RI MCA versus PI MCA	502	0.96	< 0.001

Abbreviations: MCA, middle cerebral artery; PI, pulsatility index; RI, resistance index; UA, umbilical artery; UAt, uterine artery.

The CPR percentile was normal in 476 of 502 (94.8%) and altered in 26 of 502 (5.2%) examinations. In contrast, U/C was normal in 498 of 502 (99.2%) and altered in 4 (0.8%) examinations. Of the 502 Doppler examinations, there was an agreement between U/C and CPR percentiles in 480 (95.6%) and disagreement in 22 (4.4%) examinations ( Table 3 ). In all the cases of disagreement, U/C was normal, and the CPR percentile was altered. The estimated Kappa coefficient of agreement was 0.26 with 95%CI (0.05–0.46), thereby corresponding to a weak agreement.

**Table 3:** Description of the percentile of cerebroplacental ratio by the percentile of umbilical–cerebral ratio

Percentile CPR	U/C		Total
	Normal (≤ 1)	Altered (> 1)	
Normal (≥ 5 ^th^ )	476	0	476
	94.8%	0.0%	94.8%
Altered (< 5 ^th^ )	22	4	26
	4.4%	0.8%	5.2%
Total	498	4	502

Abbreviations: CPR, cerebroplacental ratio; U/C, umbilical–cerebral ratio.

Of the 68 cases with EFW ≤ 9 ^th^ percentile (SGA), there was an agreement between U/C > 1.0 and CPR < 5 ^th^ percentile for 61 (88.4%) and disagreement in 7 (5.8%) Doppler examinations. In all cases of disagreement, the U/C > 1.0 was “no” and the CPR < 5 ^th^ percentile was “yes” ( Table 4 ). The estimated Kappa coefficient of agreement was 0.49 with 95%CI (0.13–0.85), thereby corresponding to a moderate agreement.

**Table 4:** Analysis of agreement between CPR (< 5th) and U/C (> 1.0) in fetuses with estimated weight ≤ 9 ^th^ percentile

CPR (< 5 ^th^ )	U/C (> 1.0)		Total
	No	Yes	
No	57	0	52
	89.1%	0.0%	
Yes	7	4	16
	10.9%	100%	
Total	64	4	68

Abbreviations: CPR, cerebroplacental ratio; U/C, umbilical–cerebral ratio.

## Discussion

Although FGR is one of the greatest challenges in obstetrics, there is still no treatment that can reverse placental insufficiency. In this context, the management of these patients is of fundamental importance. Additionally, Doppler examination is crucial in assessing the fetal well-being and deciding the moment of delivery while examining the risks of prematurity with the risks of fetal death.

It is important to perform fetal Doppler examination with the correct technique. Similarly, its interpretation is also important along with the knowledge of the most appropriate Doppler parameter (PI or RI) to attest the fetal vitality. In our study, we obtained an excellent correlation between the PI and RI of UA, MCA, and UtA; hence, both PI and RI can be used in the follow-up of FGR. This result is consistent with studies in the literature such as the one by Khanduri et al., 13 who concluded that both the PI and the RI of the UA have a similar accuracy for the diagnosis of FGR. Another study, conducted by Rani et al., 14 showed that the PI and RI of both UA and MCA had a similar accuracy to predict adverse perinatal outcomes in pre-eclampsia.

Also corroborating with our study, Cnossen et al. 15 conducted a systematic review with meta-analysis demonstrating that, in the 2 ^nd^ trimester, both the PI and RI of the UtA present a similar performance for the prediction of FGR (positive likelihood ratio for RI = 2.4 and for PI = 2.3) in the high-risk pregnant women.

Cerebroplacental ratio is a new Doppler tool that has recently been gaining prominence in the monitoring of FGR, so much so that it has been included in the recent classification of expert consensus based on the Delphi method to assist in the diagnosis of late-onset FGR. 14 Cerebroplacental ratio has been shown to be more sensitive to hypoxia than its individual components and demonstrates a better correlation with adverse perinatal outcomes in SGA or FGR. Triunfo et al. 16 showed that CPR improves the prediction of adverse perinatal outcomes compared with only the EFW in low-risk pregnancies at 37 weeks. Morales-Roselló et al. 17 evaluated 891 fetuses between 34 and 41 weeks and concluded that CPR was the parameter that best predicted adverse perinatal outcomes at the end of pregnancy.

There are some references for the use of this Doppler tool, and two of them were studied: the CPR, which is the ratio of the PI of the MCA divided by the PI of the UA, and the U/C, which is the ratio of the RI of the UA divided by the PI of the MCA. The data from our study showed that, for FGR cases, there was only a moderate correlation between the two parameters (kappa = 0.49) and that the use of PI would be more accurate. However, in the literature, the multicentric study PORTO 6 and the study of To et al. 11 showed that it would be possible to use both the PI and RI. The PORTO study 6 compared the CPR performed with PI and RI values to predict the adverse perinatal outcomes, and To et al. 11 compared the CPR to assess the need for an operative delivery. In both studies, the authors obtained a good correlation between the use of PI and RI. A possible explanation for this difference in our results in the relationship to previous studies may be the smaller number of FGR cases in our sample. Additionally, another possible bias was that a fixed value was used for the normality value for the U/C, whereas a reference curve variable according to the gestational age was used for CPR.

Although Doppler examination plays a very important role in FGR and can identify placental insufficiency and fetal cardiovascular adaptation, hypoxia does not yet exist as a universal concept in which an index or a reference should be used. Hence, further studies are necessary to standardize the conduct and, consequently, improve the perinatal outcomes. 18

The small number cases of FGR ( *n* = 19, 4 early- and 15 late-onset) is a limitation of the present study. Early- and late-onset FGR are two completely different entities; however, the main purpose of the present study was assessing the correlation/agreement of maternal-fetal Doppler parameters in normal and FGR fetuses. The approach to assess the maternal-fetal vessels during the Doppler examinations was the same for both early- and late-FGR as well as normal fetuses.

## Conclusion

In summary, we observed a strong correlation between RI and PI UAt, UA, and MCA Doppler; however, a weak agreement was observed between U/C and CPR in normal and FGR fetuses. In SGA fetuses, the agreement between U/C and CPR was moderate.
